# Extracellular vesicles in reproduction and pregnancy

**DOI:** 10.20517/evcna.2022.27

**Published:** 2022-09-30

**Authors:** Tahlia I. Smith, Ashley E. Russell

**Affiliations:** ^1^Department of Biology, School of Science, Penn State Erie, The Behrend College, Erie, PA 16563, USA.; ^2^Magee Womens Research Institute - Allied Member, Pittsburgh, PA 15213, USA.; **^#^** These authors contributed equally.

**Keywords:** Extracellular vesicles, reproduction, pregnancy, preeclampsia, gestational diabetes mellitus, preterm birth, intrauterine growth restriction, infertility, maternal-fetal crosstalk, spermatogenesis

## Abstract

Extracellular vesicles (EVs) are small, lipid-bound packages that are secreted by all cell types and have been implicated in many diseases, such as cancer and neurodegenerative disorders. Though limited, an exciting new area of EV research focuses on their role in the reproductive system and pregnancy. In males, EVs have been implicated in sperm production and maturation. In females, EVs play a vital role in maintaining reproductive organ homeostasis and pregnancy, including the regulation of folliculogenesis, ovulation, and embryo implantation. During the development and maintenance of a pregnancy, the placenta is the main form of communication between the mother and the developing fetus. To support the developing fetus, the placenta will act as numerous vital organs until birth, and release EVs into the maternal and fetal bloodstream. EVs play an important role in cell-to-cell communication and may mediate the pathophysiology of pregnancy-related disorders such as preeclampsia, gestational diabetes mellitus, preterm birth, and intrauterine growth restriction, and potentially serve as noninvasive biomarkers for these conditions. In addition, EVs may also mediate processes involved in both male and female infertility. Together, the EVs secreted by both the male and female reproductive tracts work to promote reproductive fertility and play vital roles in mediating maternal-fetal crosstalk and pregnancy maintenance.

## INTRODUCTION

**Figure 1:**
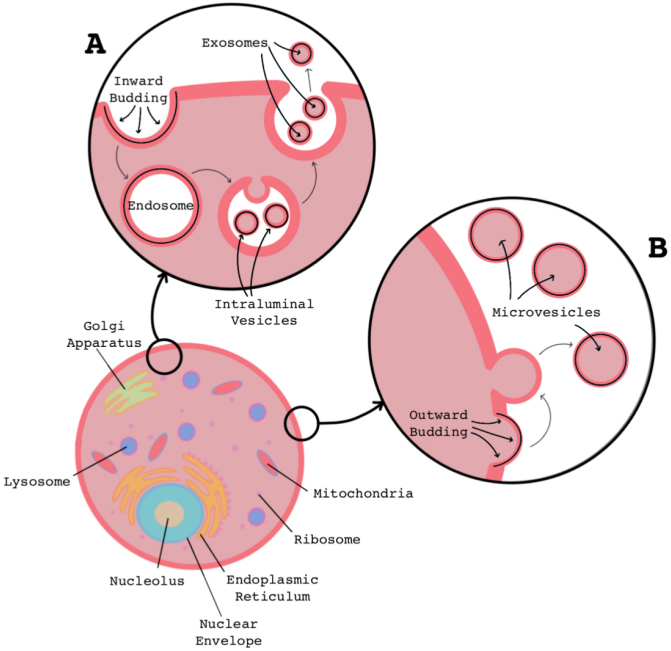
Common mechanisms of extracellular vesicle biogenesis. Formation of exosomes (A) begins with the inward budding of the cell’s plasma membrane to create an endosome. The plasma membrane of the endosome then invaginates creating intraluminal vesicles. The endosomal plasma membrane can then fuse with the cell’s plasma membrane and release the intraluminal vesicles into the extracellular space as exosomes. Microvesicles (B) are formed as the cell’s plasma membrane blebs outwards and sheds, releasing vesicles into the extracellular space.

Extracellular vesicles (EVs) are small, lipid bound packages [Figure 1] that are secreted by all cell types and are thought to play a role in both normal, homeostatic mechanisms, and several diseases including cancer^[1]^, autoimmune disorders^[2]^, and neurodegenerative disorders^[3,4]^. These particles are naturally released as a form of intercellular communication^[5]^ and can be found in all biological fluids, including saliva, urine, cerebral spinal fluid, and intravascular fluids. They function to carry bioactive molecules like RNA and DNA^[6-8]^, amino acids, lipids, and metabolites throughout the body^[9]^, and have been shown to influence various regulatory mechanisms such as skin cell development^[10]^, immune system activation^[11-13]^, and antitumor responses^[14]^.

Intercellular communication via EVs can contribute to both physiological and pathological changes in target cells^[15]^ as they play a major role in homeostasis and various diseases, such as cancer and neurodegenerative disorders^[16,17]^. Cancer is characterized by cells that divide uncontrollably and develop the ability to destroy normal body tissues. EVs have been involved in metastatic seeding which occurs when secondary tumors develop in tissues other than the tissue the cancer originated in^[18]^. EVs have also been implicated in several neurodegenerative diseases such as Parkinson’s disease, prion disease and Alzheimer’s disease^[16,19]^. In these cases, EVs contain pathological cargo and contribute to the spread of pathology from their cells of origin, into the extracellular environment^[15,20,21]^ and to both neighboring and distal cells. For example, EVs injected into the tail vein of mice were eventually observed in the lungs, spleen, bone marrow, and liver^[22]^, indicating that EVs originating in one location can influence cells in other areas of the organism.

## INVOLVEMENT IN THE REPRODUCTIVE SYSTEM

Part of maintaining homeostasis includes the maintenance of the reproductive organs, however, the role of EVs within this system is still under intense investigation.

### Extracellular vesicles in the male reproductive system

**Figure 2:**
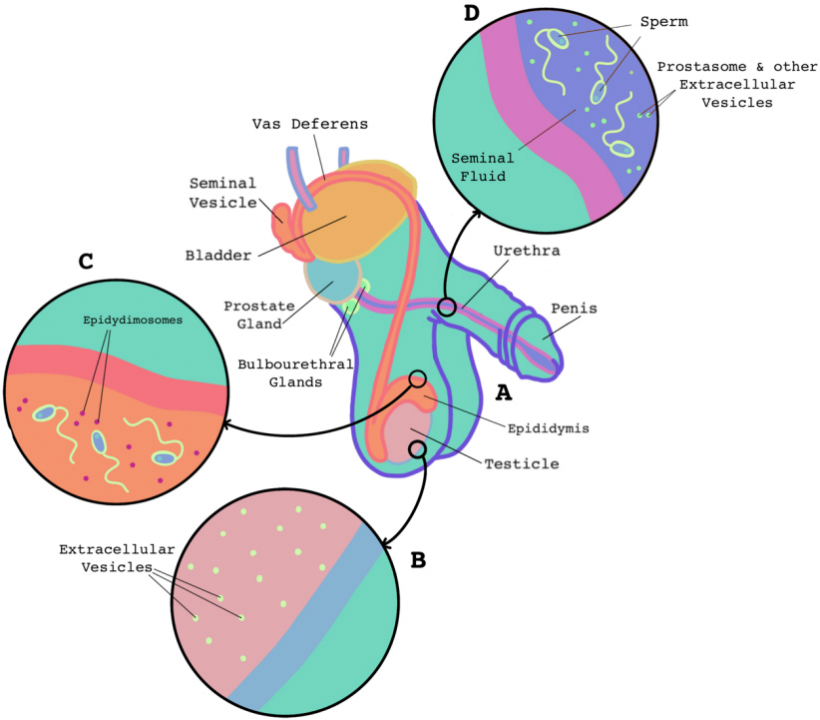
Anatomical structure and location of EVs in the male reproductive system. The primary components of the male reproductive tract (A) consist of the testes where sperm are made, the epididymis where sperm mature, the vas deferens which transports sperm to the seminal vesicles and prostate gland which produce seminal plasma, the urethra, and the penis. EVs have been identified in the testes (B) and may influence sperm production. EVs found in the epididymis (C) are referred to as epididymosomes. EVs in seminal fluid are produced by the seminal vesicles and prostate and are mixed with sperm as it enters the urethra (D). EVs: Extracellular vesicles.

The male reproductive system consists of the testes, the epididymis, accessory glands, and the penis^[23] ^[Figure 2A]. Sperm are produced within the testes and begin to mature as they move through the epididymis where they are stored. Prior to ejaculation, sperm move from the epididymis to the vas deferens, which join with the seminal vesicles and prostate gland. Together with the bulbourethral glands, the seminal vesicles and prostate produce the seminal fluid that sperm mix with to produce semen. EVs have been shown to be associated with sperm development and maturation at various locations within the male reproductive tract.

#### In the testes

Sperm are continuously produced by spermatogonial stem cells (SSC) within the testes’ seminiferous tubules^[24]^. The stem cell niche plays a key role in SSC proliferation and EVs released from developing sperm cells can suppress the proliferation rates of these SSCs, potentially serving as negative regulators of spermatogenesis^[25]^. Additionally, Sertoli cells within the seminiferous tubules have also been reported to potentially release EVs, which may impact spermatogenesis as well^[26]^. Testicular EVs [Figure 2B] have also been shown to be taken up by developing sperm cells at various points in spermatogenesis, and by epithelial cells within the seminiferous tubules, likely serving as important intercellular communicators^[27]^. The majority of studies examining male reproductive tract EVs however, focus on those produced in the epididymis or within seminal fluid.

#### In the epididymis

Sperm are produced in the testes and as they move through the epididymis, they mature and acquire the ability to fertilize an ovum. EVs located in the epididymis, also known as epididymosomes [Figure 2C], were first observed in hamsters^[28]^ and have now been found in other mammals including mice^[29,30]^, rats^[31]^, bulls^[32]^, rams^[33]^, and humans^[34]^. The protein cargo found in epididymosomes typically includes various enzymes, as well as adhesion, structural, and trafficking molecules and can act as a vehicle for modulating the molecular structure of sperm^[28,34-37]^. More specifically, epididymosomes carry proteins including, but not limited to, sorbitol dehydrogenase, hexokinase 1, acrosin, and zona pellucida binding protein 1 and 2 that interact with receptors located on the head of the sperm and mediate processes associated with maturation, protection, and the acrosome reaction^[38,39]^.

Interestingly, epididymosomal cargo varies depending on which segment of the epididymis they are isolated from^[35,36,40]^. Microarray data suggest that miRNAs are differentially expressed in cauda versus caput epididymosomes, but a large majority of these miRNAs are expressed at similar levels in the epididymal epithelial cells from which they are derived^[40]^. Conversely, small RNA sequencing data reveal that epididymosomes isolated from the cauda epididymal segment have a more complex miRNA profile relative to caput epididymosomes, and these miRNA profiles differ from those observed in the epididymal epithelial cells from which they are derived^[36]^. These disparate findings may be attributed to a number of methodological differences in sample processing, the techniques used for vesicle and RNA isolations, and how miRNAs are profiled (microarray *vs.* sequencing). Species differences may also impact epididymosomal miRNA profiles.

As sperm travel through the segments of the epididymis, their miRNA signature changes, and they acquire various capabilities such as ample motility and the ability to fertilize the female ovum^[41,42]^. Sperm are not able to perform de novo synthesis of proteins or RNAs, so many of these changes can be attributed to the uptake of epididymosomes^[40,43-46]^.

Arrdc4 is important for sperm cells to develop fertilization capacity within the epididymis^[47]^. Arrdc4 is a member of the α-arrestin protein family and plays a role in EV biogenesis^[48]^. When compared to *Arrdc4^+^/^-^* cells, *Arrdc4^-^/^-^* epididymal epithelial cells exhibited significantly reduced epididymosome production and interestingly, Arrdc4 knockout mice produce sperm with impaired functionality^[47]^. Impaired sperm functionality could be rescued by incubating *Arrdc4^-^/^-^* sperm with wild type epididymosomes, indicating that Arrdc4 is critical for both normal epididymosome biogenesis and sperm maturation^[47]^.

In addition to facilitating sperm maturation, epididymosomes also appear to be the main source of non-coding RNAs found in sperm, and these RNAs can alter the epigenetic inheritance patterns of offspring^[49-53]^. Epididymosomes may also play an important role in quality control of sperm cells as well by transferring epididymal sperm binding protein 1 (ELSPBP1) to dead sperm cells, possibly to protect viable sperm in the vicinity from dead or dying sperm^[54]^.

#### In the seminal vesicles and prostate

Prior to ejaculation, sperm travels up the vas deferens and mixes with the seminal plasma produced by the seminal vesicles and prostate gland to create semen [Figure 2D]. Seminal plasma consists of sugars, salts, amino acids, and other compounds necessary to maintain sperm viability. EVs have also been observed in seminal fluid since the late 1960’s, however their main physiological function is still not yet fully understood^[55]^. Commonly, seminal fluid EVs are referred to as prostasomes, which refers to a specific type of EV released from prostate epithelial cells; however, EVs in seminal fluid are highly heterogeneous and are likely derived from a number of cell types within the male reproductive tract^[56-59]^.

Seminal fluid EVs are thought to play a number of important roles in sperm functionality including forward sperm motility, capacitation, acrosomal reaction, and membrane stabilization^[60-68]^. Interestingly, prostasomes have been shown to inhibit spontaneous capacitation and acrosomal reactions by decreasing spermatozoa tyrosine phosphorylation^[65]^, and only after capacitation has been initiated do they fuse to sperm^[64]^. Indeed, it seems that seminal fluid EVs only interact with sperm after they have been introduced to the female reproductive tract^[69]^. Proteomic analysis of prostasomes revealed over 139 proteins, including prostate-specific antigen and prostatic acid phosphatase^[70]^. One third of the identified proteins were categorized as enzymes suggesting prostasome cargo may have the capacity to influence a cell’s metabolic state, while the remaining proteins function primarily as transport and structural proteins, GTP proteins, chaperones, and signal transduction proteins^[70]^. 

Semen is highly enriched in EVs containing a variety of small RNAs, including miRNA, tRNA, Y RNAs, and fragments of mRNAs^[71]^. In humans, the most abundant miRNAs were from the let-7 family, followed by miR-148a, miR-375, and miR-22; these miRNAs all have validated immune-related mRNA targets, suggesting that they may modulate the immune function of semen in the female reproductive tract^[71]^. In boar semen, miR-21-5p, miR-148a-3p, miR-10a-5p, miR-10b, miR-200b, and the let-7 family are the most abundant EV-associated miRNAs^[72]^. Interestingly, EV-associated miR-21-5p has been found to potentially reduce sperm fertility, and along with miR-148a and the let-7 miRNAs, may impact immune function^[72]^.

### Extracellular vesicles in the female reproductive system

**Figure 3:**
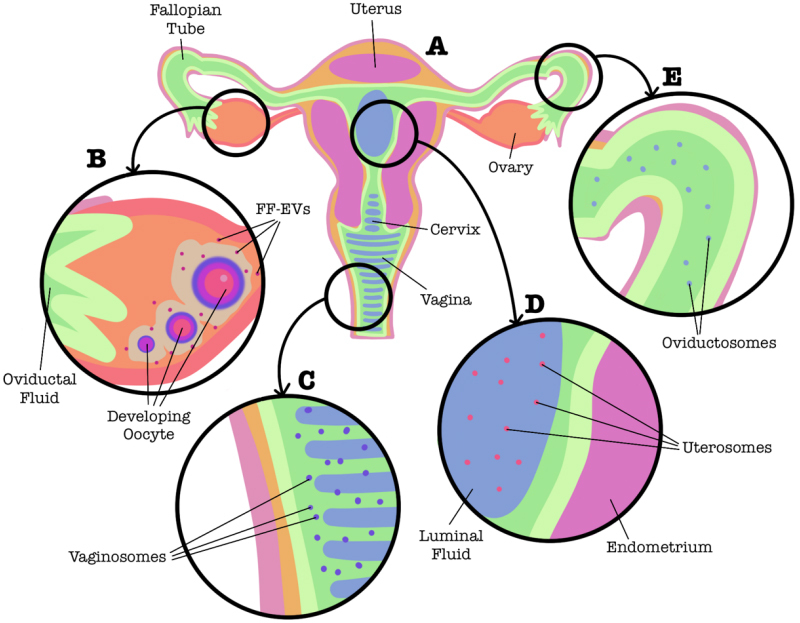
Involvement of EVs in the female reproductive system (A). In female organisms of many species, secretions from the epithelial lining of the ovary (B), cervix and vagina (C), endometrium (D), and fallopian tubes (E) contain EVs. EVs: Extracellular vesicles.

The female reproductive system consists of the ovaries, fallopian tubes, uterus, and vagina [Figure 3A]. Within the ovary, eggs are surrounded by follicular fluid. During ovulation, an egg is released from the ovary and brought into the fallopian tube, filled with oviductal fluid. The egg then travels down the fallopian tube to the uterus where, if fertilized, it will become implanted into the uterus and begin to develop into an embryo. 

The role of EVs in the female reproductive system is heavily understudied but is beginning to gain traction, especially in livestock like cows, horses, and sheep. In humans, EVs have been detected in follicular fluid, oviductal fluid, the intrauterine environment, and in the vagina [Figure 3].

#### In the ovaries

Follicular fluid (FF) is the liquid content that surrounds the developing oocyte, the cell in an ovary that has the potential to form an ovum [Figure 3B]. Within the ovary, mural granulosa cells line the follicle and communicate with the cumulus-oocyte complex (COC; specialized granulosa cells that surround and support the oocyte). Prior to ovulation, the COC expands, allowing for the oocyte to undergo complete meiotic maturation and likely occurs due to communication between granulosa cells and the COC^[73]^. EVs within the FF (FF-EVs) have been shown to induce COC expansion and this mechanism may be mediated by miRNAs within these EVs^[74,75]^. Indeed, FF-EVs have been shown to be taken up by granulosa cells, which further supports the notion that these EVs impact follicle growth and development^[76]^. Human derived FF-EVs were previously shown to contain miRNAs that target genes associated with the inhibition of follicular maturation and the resumption of meiosis, including miR-132, miR-212, and miR-214^[77]^. Conversely, another group demonstrated that FF-EVs arrest meiotic maturation of oocytes through the CNP-NPR2 signaling pathway^[78]^. These differences in EV mediated effects may be attributed to changes in their molecular cargo at differing times in the menstrual cycle. Indeed, several studies identified significant changes in EV cargo at different phases of the menstrual cycle, suggesting that FF-EVs and their cargo are dynamic players in folliculogenesis^[79-81]^.

The FF is a rich source of EVs and through proteomic profiling, hundreds of proteins have been identified^[76,82,83]^. Many of these proteins are associated with protein and RNA folding, molecular transport, and signal transduction, and likely play important roles in oocyte competence and follicular homeostasis^[82]^. Small RNA sequencing of extracellular RNA isolated from FF reveals the presence of numerous types of noncoding RNAs, including miRNA, snRNA, snoRNA, and tRNA^[84,85]^. Separation of EVs from the FF supernatant reveal significant overlap in expression of several miRNAs that target genes involved in reproduction, cell proliferation, and immune system development^[84]^. The mRNA profiles contained within FF-EVs have also been sequenced, and many of the identified sequences code for proteins involved in metabolic pathways, DNA-protein interactions, and transcriptional regulation^[85,86]^.

Proper signaling of the transforming growth factor-β (TGF-β) pathway is required for follicular development and oocyte competence^[87]^. FF-EVs from horses have been shown to contain ACVR1 (a member of the TGF-β superfamily) mRNA and protein, as well as miRNAs that regulate expression of ACVR1, including miR-27b, miR-372, and miR-382^[76,88]^. Porcine and bovine FF-EVs were also found to contain miRNAs that target genes involved in the TGF-β signaling pathway^[89,90]^. These data further support the notion that FF-EVs participate in mediating follicular development and homeostasis. 

FF-EVs have also been shown to be taken up by and alter the transcriptome of epithelial cells that line the fallopian tubes, leading to the expression of genes that would assist in increasing the likelihood of fertilization and embryo development^[91]^.

#### In the fallopian tubes

The fallopian tubes, or oviducts, are filled with fluid produced by secretions from epithelial cells that line the oviducts and the transudation from the blood [Figure 3E]. This oviductal fluid also contains EVs which are referred to as oviductosomes^[92]^. 

Oviductosomes have primarily been studied within the context of fertilization as they have been shown to transfer key proteins to sperm within the female reproductive tract (discussed later in "Extracellular vesicles and fertilization" below). Proteomic analysis of feline oviductosomes revealed over 1000 proteins, many of which are involved in metabolism and cellular organization^[93]^. The bovine oviductosome proteome was less diverse with slightly over 300 proteins identified, however gene ontology analysis revealed the majority of these proteins were also involved in metabolism and localization^[94]^.

The protein and RNA cargo of bovine oviductosomes has been shown to fluctuate throughout the course of the estrous cycle^[95]^. Similar findings were observed for murine oviductosome miRNA and metabolomic profiles as well^[96,97]^. These data suggest that oviductosome composition is under hormonal regulation.

#### In the uterus

Extracellular vesicles found in the luminal fluid of the uterus [Figure 3D] are commonly referred to as uterosomes^[98]^. The protein and RNA cargos of these vesicles differ from the endometrium lining, and change during pregnancy^[99,100]^. Expectedly, the estrous cycle also affects the release and cargo composition of uterosomes as well^[100-102]^. Uterosome associated proteins released from endometrial epithelial cells appear to be involved in key embryo-implantation mechanisms, indicating that these vesicles play important roles in early pregnancy^[102]^. Aside from being released from endometrial cells, it has also been shown that uterosomes can be taken up by endometrial epithelial cells and significantly alter their transcriptome^[103]^.

#### In the vagina

Very recently, EVs present in the vaginal canal have begun to be examined [Figure 3C]. Termed vaginosomes, these vesicles released from the vaginal lining have been shown to modulate sperm capacitation and acrosome reaction in mice, similar to EVs found in other female biofluids^[104]^. When assessing the miRNA profiles of vaginal fluid however, the majority of miRNAs identified were non-vesicular^[105]^. Interestingly, there is some evidence that suggests extracellular RNAs found in the vagina can protect against HIV-1 infection, specifically miR-186-5p^[105]^. Although not released directly from the vaginal epithelium, there is evidence to suggest that bacterial-derived EVs found in the vaginal compartment also provide protection against HIV-1 infection^[106,107]^.

## ROLES OF EXTRACELLULAR VESICLES IN NORMAL PREGNANCY

### Extracellular vesicles and fertilization

When an egg is released, it is transported to the fallopian tube where it may be fertilized. As discussed previously, the fallopian tubes contain oviductal fluid and oviductosomes, which have been shown to play a number of different roles in mediating fertilization, including sperm capacitation, the acrosome reaction, gamete maturation, and embryo quality.

Sperm capacitation, also known as sperm activation, occurs in the female reproductive tract and initiates the signaling pathways necessary for the sperm to penetrate the multiple layers of the female egg, including alterations to the sperms metabolism, membrane structure and permeability, and pH^[108,109]^. In the first step of fertilization, the sperm’s plasma membrane binds to the outer layer of the oocyte; the zona pellucida. After this binding event, the acrosome reaction occurs, in which the head of the sperm releases hydrolytic enzymes to soften the zona pellucida, allowing for further entry into the oocyte. Lastly, the cortical reaction occurs when the female egg acknowledges the presence of the male sperm and releases cortical granules to harden the zona pellucida to prevent polyspermy, and the sperm’s genetic information is deposited inside of the oocyte. 

During sperm capacitation, sperm cells acquire plasma membrane Ca^2+^-ATPase 4a (PMCA4a) and PMCA1 from oviductosomes, uterosomes, and vagisomes^[92,104,110]^. This mechanism plays an essential role in fertilization as post-testicular acquisition of these calcium pumps is paramount for sperm motility and the acrosome reaction^[92]^. Oviductosomes and uterosomes have also been shown to transfer tyrosine phosphorylated proteins to sperm, which may also modulate capacitation as this is a key signaling event that occurs in capacitation^[98,110]^.

In addition to proteins, miR-143-3p, miR-22-3p, and miR-34c-5p were also shown to be transferred from oviductosomes to sperm cells, and were localized to sperm heads^[96]^. Most interestingly, miR-34c was localized near the centrosome where it has previously been shown to play a key role in the first cleavage division of the fertilized egg^[111]^.

Incubation of sperm with oviductosomes improved sperm viability and motility in a dose and time-dependent fashion, including sperm from frozen semen which holds great promise for improving *in vitro *fertilization (IVF) outcomes^[93,112]^. Aside from being taken up by sperm, oviductosomes have also been shown to be taken up by oocytes and participate in the regulation of monospermy, potentially by delivering the oviduct-specific protein OVGP1, which has previously been shown to help prevent polyspermy, a key issue with IVF^[112-114]^. Additionally, uterosomes have been shown to transfer Sperm Adhesion Molecule 1 (SPAM1) to sperm, which is a crucial protein involved in the fertilization process^[30]^. Uterosome EVs taken up by sperm cells have been shown to stimulate capacitation as well^[115]^. 

IVF requires the addition of sperm to an extracted oocyte, however the oocyte needs to fully mature prior to fertilization. Several extracellular miRNAs isolated from the FF of oocytes retrieved from women undergoing IVF were identified to be indicative of high fertilization potential; specifically miR-202-5p, miR-206, miR-16-1-3p, and miR-1244^[116]^. MiR-92a and miR-130b were found to be significantly upregulated in FF-EVs derived from oocytes that failed to be fertilized^[117]^. Incubation of retrieved oocytes with FF-EVs and/or oviductosomes has been shown to improve oocyte maturation and embryonic development^[85,118,119]^. Interestingly, the method by which FF-EVs or oviductosomes are isolated from follicular or oviductal fluid impacts IVF-embryonic competence, with vesicles isolated via OptiPrep^TM^ density gradient ultracentrifugation yielding higher quality blastocysts than those isolated via size exclusion chromatography^[118]^.

### Embryo implantation and maternal-fetal crosstalk

After fertilization, the conceptus trophectoderm releases EVs into the uterine fluid and these vesicles likely mediate some communication between the endometrium lining and the fertilized egg prior to implantation^[99,120,121]^. Interestingly, embryonic stem cells within the inner cell mass of the embryo also release EVs and promote implantation of the blastocyst^[122]^. Seminal fluid EVs present within the female reproductive tract may also play a role in mediating endometrial decidualization and promoting prolactin secretion, both of which are necessary for embryo implantation^[123]^. 

The endometrium releases uterosomes which are taken up by the embryo prior to implantation, and notably, EV-associated miR-30d increases the adhesion of the embryo to the endometrium^[124]^. Hormonal signaling also influences the cargo of uterosomes and can impact the adhesive capacity of embryos through focal adhesion kinase (FAK) signaling^[102]^. MiR-100-5p was identified in EVs isolated from endometrial cells and has also been shown to activate FAK signaling and promote embryo implantation^[125]^. Other miRNAs identified in the conditioned cell culture media of endometrial cells include miR-200c, miR-17, and miR-106a, all of which are involved in pathways associated with embryo implantation^[121]^. The bidirectional communication of the endometrial and trophoblast cells is mediated in part by EVs, allowing for the transfer of important cargo to facilitate embryo implantation, such as angiogenic and proliferation factors^[126]^.

The number and size of EVs released from IVF embryos may be indicative of embryo quality as recent evidence suggests that lower quality embryos release more EVs relative to higher quality embryos, and these EVs are slightly smaller in diameter on average^[127-129]^. Further, the small RNA cargo of blastocyst derived EVs may also be indicative of quality and viability as some evidence suggests there may be specific miRNA profiles that are up- or down-regulated in EVs released from viable/non-viable embryos^[130]^.

Co-culturing IVF embryos creates a microenvironment that relies on paracrine communication and results in improved embryonic development compared to embryos cultured independently. This phenomenon may be due in part to EVs released from the embryos that contain the pluripotency genes *Nanog*,* Klf4*,* Oct4*,* Sox2*, and* c-Myc*, which improve the developmental competence of co-cultured, neighboring embryos^[131]^. Together these data may help improve IVF outcomes by creating optimal culturing environments and improving identification of the most promising embryos. 

### Placental derived extracellular vesicles

As the embryo makes itself at home in the uterine lining, it must continue to communicate with the surrounding environment to ensure that the maternal immune system does not reject it, as this could result in spontaneous abortion. Early on in pregnancy, maternal immune tolerance during implantation is typically mediated by regulatory T cells which function to inhibit inflammatory responses and allow for implantation of the developing embryo.

**Figure 4:**
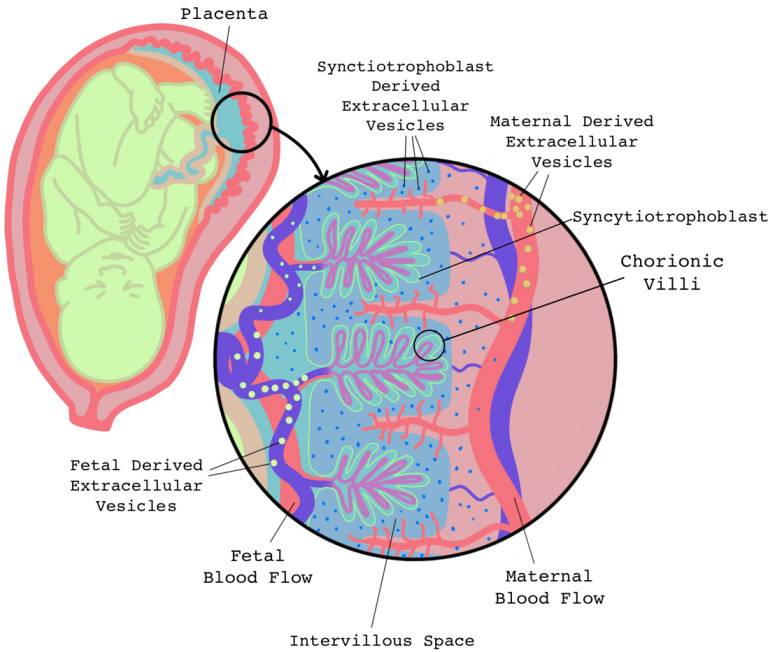
Maternal-fetal communication through EVs. The placenta acts as a central hub throughout the duration of the pregnancy. During this time, the fetus, placenta, and maternal cells release EVs that facilitate communication between the maternal environment and the fetus. EVs: Extracellular vesicles.

The placenta is an organ that develops during pregnancy and serves as the main form of communication between the mother and the growing fetus [Figure 4]. The placenta attaches to the mother’s uterus between weeks 10 and 12 in humans, through remodeling of the uterine wall spiral arteries, mediated by extravillous cytotrophoblasts. The placenta sustains the fetus for several months, serving as the main transporter of oxygen and nutrients for the developing fetus^[132]^.

As the placenta begins to develop, this transient organ works to modulate the maternal immune response to ensure that the maternal immune system does not reject the developing fetus; this also allows the placenta to interface and communicate with the maternal circulatory system^[133]^. Some of this communication is thought to be mediated by EVs released from the placenta^[134,135]^. Indeed, EVs released from cytotrophoblasts have been shown to be involved in the migration of extravillous trophoblasts into the decidua of the uterus^[136]^. 

Placental derived EVs are typically thought to express placental alkaline phosphatase (PLAP) and have been shown to increase in maternal circulation throughout pregnancy, beginning in the first trimester^[137]^. Placental derived EVs have been shown to express GLA-G5, B7-H1, and B7-H3, which are immunomodulatory proteins that can modulate T cell responses and may be implicated in maternal-fetal tolerance^[138]^. Additionally, placental derived EVs have been shown to express Fas ligand (FasL) and induce FasL-mediated apoptosis in T cells, which also assists in maternal-fetal tolerance^[139,140]^. Placental derived EVs also carry ligands for the natural killer cell receptor, NKG2D, including UL-16 binding proteins (ULBPs), and MHC class 1 chain-related (MIC) proteins A and B^[141]^. These proteins participate in the fetal immune escape by reducing natural killer cell cytotoxicity^[141]^. 

As the placenta develops, it invades the uterine decidua and induces reorganization of the uterine spiral arteries. Recent evidence suggests that placental expression of miR-18a, miR-518b, and miR-376c promote trophoblast invasion of the decidua, whereas miR-34a suppresses invasion^[142-145]^. Although some of these miRNAs have been observed in other tissues, miRNA profiling of placental tissue revealed a cluster of primate-specific miRNAs, chromosome 19 microRNA cluster (C19MC), which is one of the largest miRNA gene clusters found in humans and is predominately expressed in placental tissues^[146]^. Interestingly, C19MC miRNAs are of the most abundant miRNAs identified in placental derived EVs both *in vitro *and *in vivo*^[147,148]^. 

MiRNAs within the C19MC have been shown to play important roles in pregnancy and these miRNAs have been observed in circulating EVs of pregnant women^[147,149]^. For example, miR-517a/c expressed in villous trophoblasts helps to mediate the endothelial to mesenchymal transition these cells must undergo to properly invade the decidua^[150]^. EV-associated miR-519c was shown to be a placental-specific immune regulator and may have anti-inflammatory properties^[151]^. Trophoblast cells confer resistance to maternal viruses via expression of miRNAs in the C19MC, specifically miR-517-3p, miR-516b-5p, and miR-512-3p, and this resistance can be transferred to nonplacental cells via EVs^[152]^. These miRNAs are detectable in maternal blood EVs as early as two weeks after implantation of IVF embryos^[153]^. In addition to the C19MC cluster, the miR-371-3 cluster (also present on chromosome 19) and the C14MC (present on chromosome 14) are also predominately expressed by the placenta and detectable during pregnancy^[154]^. 

Understanding the biodistribution of EVs during pregnancy would better elucidate their specific roles in various tissues across time. For example, recent evidence suggests that placental-derived EVs traffic to the maternal lung and liver, and specifically interact with immune cells in these organs through surface integrins^[155]^. Interestingly, when assessing distribution of large *vs.* small placental EVs, macro-vesicles were found to localize to the lungs exclusively, while micro-vesicles were found in the lung, liver, and kidneys^[156]^. Similarly, human placental EVs injected into a pregnant mouse model were found to localize to the kidney, lungs, and liver, and after a 30-min exposure period, the acetylcholine-mediated vasodilatory response of the murine mesenteric arteries was increased; however, after 24 h this effect was reversed^[156]^. Through use of a transgenic mouse line, it was shown that just over a third (35%) of maternal plasma EVs are of fetal origin and are also localized to the maternal uterine environment^[157]^. Further, injection of bioengineered EVs into pregnant mice showed trafficking of EVs to fetal cells as well, suggesting that maternal EVs can cross the placenta and influence the fetus^[157]^.

## ROLES OF EXTRACELLULAR VESICLES IN PREGNANCY COMPLICATIONS

Much of the research focused on placental derived EVs focuses on how they may be used as diagnostic tools or biomarkers for various pregnancy complications as many women throughout the United States experience complications with pregnancy every day^[158]^. Usually, these complications are health problems that occur during pregnancy and can impact either the health of the mother, the baby, or both. Some of these issues can be present before the pregnancy begins but are exacerbated by it, like hypertension or anemia^[159]^. Others, such as preeclampsia and gestational diabetes, arise after the woman becomes pregnant. Table 1 describes the cellular origin and potential function of these EVs in preeclampsia, gestational diabetes mellitus, preterm birth, and fetal growth restriction.

**Table 1:** Cellular origin and potential function of EVs involved in the pathogenesis of various pregnancy related diseases

**Pregnancy condition**	**Cellular origin**	**Potential function**	**Citation**
Preeclampsia	Placenta	Reduced expression of syncytin-2, which alters syncytiotrophoblast formation	[160]
	Placenta	Alter fibrinolytic and angiogenic processes	[161]
	Placenta	Activation of peripheral blood mononuclear cells and induce proinflammatory response	[162]
	Trophoblast cells	Stimulate EV release from endothelial cells	[163]
	Syncytiotrophoblasts	Activate platelets, promote coagulation	[164]
	Endothelial cells	Express high-mobility group box 1 and promote coagulation and neutrophil activation	[163]
	Platelets	Promote coagulation	[165,166]
	Podocytes	May be involved or indicative of renal injury	[167]
	N/A	Alter sodium reabsorption in the kidney	[168]
Gestational diabetes mellitus	Placenta	Induce inflammation from endothelial cells	[169,170]
		Mediate skeletal muscle insulin sensitivity	[171]
		Alter metabolic pathways associated with GDM	[172]
	N/A	Mediate glucose intolerance during pregnancy	[173]
		Insulin secretion and regulation; Glucose transport	[173,174]
Preterm birth	Placenta	Biomarkers of placental function	[175]
	Amnion epithelial cells	Increase inflammation in maternal uterine cells	[176]
		Carry HMGB1; Induce labor	[177]
	Group B *Streptococcus*	Induce labor	[178]
	N/A	Biomarkers of preterm labor	[179-181]
Fetal growth restriction	Placenta	Mediate maternal immune tolerance to the fetus	[140,182]
	Umbilical cord blood	Reduce angiogenic properties of human umbilical vein endothelial cells	[183]

EVs: Extracellular vesicles; GDM: gestational diabetes mellitus.

### Extracellular vesicles and preeclampsia

Preeclampsia is a hypertensive pregnancy disorder distinguished by the development of high blood pressure and proteinuria at around twenty weeks of gestation, as well as poor placentation and endothelial dysfunction^[184,185]^. There are several risk factors that may contribute to the development of preeclampsia, such as the mother’s age, weight, and current health status^[186]^.

**Figure 5:**
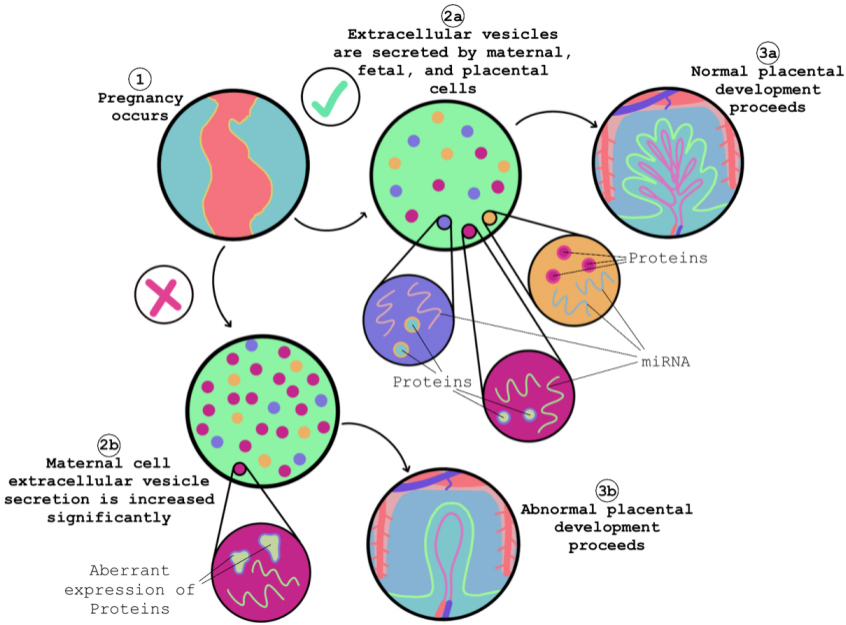
EVs released by various cell types during pregnancy may contribute to the etiology and pathophysiology of preeclampsia by transferring various RNAs and proteins that negatively impact placental development and implantation. EVs: Extracellular vesicles.

Numerous groups have demonstrated that the concentrations of circulating EVs are significantly higher in pregnant women versus nonpregnant women, and even more so in preeclamptic and eclamptic women relative to normotensive pregnant women^[137,187-189]^. Although the etiology of preeclampsia is not yet fully understood, it is thought that the process of forming new blood vessels to supply the placenta is compromised, and this may be mediated, in part, by EVs [Figure 5]. Syncytin-2, a protein that facilitates embryo implantation and trophoblast cell fusion, is expressed at lower levels in EVs isolated from preeclamptic women relative to their normotensive counterparts^[160,190]^. Cytotrophoblast cells are believed to release EVs that induce extravillous trophoblast cell invasion of the decidua; however hypoxia can impair this process and result in insufficient arterial remodeling^[136]^. Placental hypoxia also induces the release of high mobility group box-1 protein from trophoblast cells, which then stimulates the release of endothelial cell derived EVs^[163]^.

Interestingly, the majority of EVs identified in the circulation of preeclamptic women were found to be of endothelial origin and may contribute to the procoagulant phenotype seen in preeclampsia^[163]^. Activated platelets, signified by externalized phosphatidylserine (PS) contribute to coagulation, and activated platelet-derived EVs have recently been shown to express increased levels of procoagulant tissue factor (CD142) and PS in preeclamptic patients, relative to healthy controls^[165,166]^. Placental-derived EVs from preeclamptic women have also been shown to activate platelets, further perpetuating the procoagulant effect seen in preeclampsia^[164]^. Additionally, plasminogen activator inhibitor-1 (PAI-1) is overexpressed on EVs isolated from preeclamptic placentas, providing additional support for the notion that EVs contribute to coagulation in preeclampsia^[161]^.

In addition to the procoagulant effects observed in circulating EVs of preeclamptic women are the anti-angiogenic properties of these EVs^[191,192]^. Indeed, EVs obtained from placental perfusions exhibited increased levels of anti-angiogenic proteins (i.e. Eng and PAI-2), which may impact placental circulation^[161]^. EVs in preeclampsia may also impact immune cell function and further modulate the etiology and pathophysiology of preeclampsia^[193]^.

A recent meta-analysis examining the miRNA profiles of preeclamptic women indicated significant elevation of miR-16, miR-20b, miR-23a, miR-29b, miR-155, and miR-210 compared to controls^[194]^. Not surprisingly, many of these miRNAs are involved in pathways that regulate trophoblast proliferation and invasion, as well as angiogenesis^[194]^. When assessing EV cargo specifically, seven miRNAs (miR-153, miR-222-3p, miR-224-5p, miR-325, miR-342-3p, miR-532-5p, and miR-653-5p) were found to be significantly upregulated in preeclamptic women^[195]^. These miRNAs may contribute to the pathogenesis of preeclampsia, but further investigation is warranted. 

Early diagnosis of preeclampsia is of utmost importance to ensure the health and safety of both the mother and fetus. EVs in urine, blood plasma, and gingival crevicular fluid (GCF), which is an inflammatory periodontal exudate utilized for diagnosing periodontal disease^[196]^, are now being explored as noninvasive biomarkers for preeclampsia. GCF EVs from preeclamptic patients had significantly higher expression of the anti-angiogenic protein, soluble fms-like tyrosine kinase-1 (sFlt-1), and lower levels of the pro-angiogenic protein, placental growth factor (PlGF) compared to controls^[197]^. Notably, these GCF EVs were also positive for PLAP, indicating that they were indeed placental derived^[197]^. 

Urine is easily attainable and routinely collected during prenatal visits to assess protein and sugar levels as indicators for preeclampsia and gestational diabetes, respectively. As such, there is interest in using urinary EVs to detect and diagnose preeclampsia, which may also indicate renal injury associated with preeclampsia^[198]^. Urine from preeclamptic women has been previously shown to contain both podocytes (specialized epithelial cells that cover the outer surfaces of glomerular capillaries) and the podocyte specific proteins PARD-3 and PARD-6, which support the hypothesis that preeclampsia is characterized by podocyte loss and injury^[199,200]^. The number of EVs of podocyte origin isolated from the urine of preeclamptic women was significantly higher than in normotensive in pregnant women^[167]^. Further, preeclamptic urinary EVs were also shown to have altered expression of the NaCl2-k co-transporter 2, Na-Cl co-transporter, and the epithelial sodium channel which may increase sodium reabsorption in the kidney and perpetuate hypertension in preeclampsia^[168]^.

### Extracellular vesicles and gestational diabetes mellitus

Gestational diabetes mellitus (GDM) is associated with inadequate cell functionality and marked insulin resistance and is a form of hyperglycemia first detected during pregnancy^[201]^. GDM appears to result from the same physiological abnormalities that characterize diabetes mellitus outside of pregnancy^[202]^. In general, hyperglycemia results from an insulin supply that is too low to maintain blood glucose regulation^[203]^. During pregnancy, hyperglycemia caused by GDM can be associated with preterm delivery, low birth weight, and even clinical neonatal hypoglycemia^[204]^. EVs may participate in the pathophysiology of this disorder and also hold the potential to serve as noninvasive biomarkers^[205]^. 

Recent evidence is conflicting on EV profiles in pregnant women. Some studies suggest that the total number of circulating EVs does not change between women diagnosed with GDM and healthy controls^[206]^, while others report significantly more EVs in GDM plasma relative to controls^[169]^. These differences may be due, in part, to EV isolation and characterization techniques as Franzago *et al.*,^[206]^ profiled EVs from whole blood through flow cytometry, while Salomon *et al.*^[169]^ first separated EVs from blood plasma using a density gradient. Interestingly, Franzago and colleagues’ work demonstrates no significant differences in the number of total circulating EVs, but did find that the proportion of adipocyte-derived EVs is significantly higher in controls, relative to GDM patients^[206]^. Placental-derived EVs were shown to be at significantly higher concentrations in plasma obtained from GDM patients, relative to healthy controls^[169,173]^. These EVs were also shown to induce proinflammatory cytokine release from endothelial cells, which is in line with the hypothesis that GDM is associated with chronic, low-grade inflammation^[169,170]^. Circulating GDM EVs infused into a nonpregnant mouse model also conferred insulin resistance, providing further evidence that EVs play a key role in the pathophysiology of GDM^[173]^.

miRNA profiles of serum-derived EVs reveal significant differences in control versus GDM patients, and many of the dysregulated miRNAs are associated with insulin regulation, glucose transport, and trophoblast proliferation^[174]^. EVs isolated from chorionic villi explants from GDM and healthy controls revealed distinct miRNA profiles, and many of the altered miRNAs target carbohydrate metabolism and cellular migration pathways^[171]^. Urinary EVs also reveal differential expression of miRNA profiles in GDM patients, specifically downregulation of several C19CM miRNAs during the third trimester; these miRNAs target genes associated with insulin resistance and pro-inflammatory responses^[172]^.

### Extracellular vesicles and preterm birth

Babies born prior to 37 weeks gestation are considered preterm, and the mechanisms that initiate the transition of a resting uterus to a laboring one, known as parturition, are not fully understood. Increased inflammatory signals at the feto-maternal interface (FMi) may play a role. Amnion epithelial cells undergoing oxidative stress release EVs that significantly upregulate inflammatory marker expression from maternal uterine cells^[176]^. The inflammatory signaling marker, high mobility group box 1 protein (HMGB1) is elevated in the amniotic fluid, and umbilical cord and maternal blood in preterm births, and has recently been shown to be released from senescent amnion epithelial cells at the FMi in EVs^[177]^. Further, EVs expressing HMGB1 injected into pregnant mice induced preterm labor, indicating a key role in this EV-mediated inflammatory signaling pathway involved in the induction of preterm labor^[177]^. As gestation progresses, expression of EV-associated inflammatory mediators significantly increases^[207]^. Pregnant mice (E15) injected with EVs isolated from late-gestation plasma (E18), but not early-gestation (E9), showed increased expression of inflammatory mediators in their reproductive tracts and delivered preterm^[207]^.

Inflammation-induced preterm labor can also be mediated by infections, as vaginal infection with Group B *Streptococcus* (GBS) has previously been shown to induce preterm labor. Interestingly this phenomenon may be mediated by EVs released from the bacteria themselves^[178]^. A recent study found that EVs engineered to express IB, an NF-B inhibitor, could be used to suppress infection-induced preterm labor by reducing the fetal inflammatory response^[208]^.

To better understand the role of circulating EVs in preterm labor and investigate their potential as diagnostic biomarkers, EVs isolated from the blood plasma of pregnant women have been assessed for their lipid, protein, and miRNA content. Lipidomics revealed a panel of five EV-associated lipids that were found to be predictive of preterm birth^[179]^, proteomics identified 96 differentially expressed proteins in placental-derived circulating EVs^[175]^, while RNA sequencing identified over 150 significantly altered miRNAs in circulating plasma EVs^[180,181]^. Interestingly, a number of differentially expressed miRNAs were associated with placental development^[181]^. Together, these data support the need for further mechanistic studies and validation of the use of EV profiling for the prediction of preterm labor.

### Extracellular vesicles and intrauterine growth restriction

Intrauterine growth restriction (IUGR) occurs when a fetus’ growth is in less than expected for their gestational age^[209]^. IUGR occurs in a subset of pregnancies and usually characterized by placental insufficiency, and sometimes linked to preeclampsia^[210]^. As EVs have been implicated in the growth, implantation, and angiogenic properties of the placenta (discussed in "ROLES OF EXTRACELLULAR VESICLES IN NORMAL PREGNANCY"), it is not surprising that EVs may also play a role in IUGR. In a cohort of 30 pregnant women, 20 of which were either small for gestational age or experienced IUGR, fewer placental-derived EVs were found in maternal plasma from IUGR pregnancies compared to normal birthweight pregnancies^[211]^. An important aspect of normal pregnancy is maternal immune tolerance to the fetus, and this may be mediated, in part, due to placental derived EVs expressing FasL and other immunomodulatory molecules^[140,212]^. In IUGR pregnancies, circulating EVs from maternal plasma express lower levels of FasL relative to those from normal pregnancies, and which may result in less maternal immune tolerance of the developing fetus and impaired fetal growth^[182]^.

Another study observed the downregulation of several C19MC cluster miRNAs in EVs isolated from the blood plasma of women during their first trimester who would then go on to develop IUGR^[213]^. Further, in a porcine model of IUGR, significant differences in EV-associated miRNAs associated with angiogenesis, specifically decreased expression of miR-150, were observed and may contribute to abnormal placentation in IUGR pregnancies^[183]^. Similar to IUGR, studies on small for gestational age pregnancies also show altered expression in EV-associated miRNAs, with increased expression of miR-20b-5p, miR-942-5p, miR-324-3p, miR-223-5p, and miR-127-3p^[214]^. The role of EVs in mediating the pathophysiology of IUGR is just beginning to be elucidated and warrants further mechanistic studies.

### Extracellular vesicles and infertility

Currently, infertility is defined as one year of unwanted non-conception with unprotected intercourse in the fertile phase of the menstrual cycle^[215]^. After six cycles of attempted conception, about 50% of the couples trying to conceive will do so spontaneously in the next six cycles and the remaining would be considered as having slightly reduced fertility^[215]^. After twelve unsuccessful cycles, 50% of these couples will conceive in the next 36 months (about 3 years) while the rest are nearly completely infertile; by forty-eight cycles of attempted conception, couples are equivalent to sterile^[215]^.

In cases with a more positive prognosis, most couples are encouraged to wait for any assisted reproductive treatment because their probability of conceiving with or without treatment is very much the same^[216,217]^. Most of the time, self-monitoring of the reproductive system is recommended and may be all that is necessary to improve the chances of conception^[218,219]^. However, cases with a more negative prognosis, such as tubal pathology or severe male infertility, immediate assisted reproductive treatment should be discussed because it would increase the chances of conception rather than with self-monitoring^[220]^.

#### Male infertility

Male infertility is typically characterized by low sperm count (oligozoospermia), low sperm motility (asthenozoospermia), both low sperm counts and motility (oligoasthenozoospermia), or no sperm in the semen at all (azoospermia). Extracellular RNAs have been posited to play a role in male infertility and recent evidence suggests that alterations in their expression may be indiciative of the different types of infertility and potentially be involved in the underlying mechanisms associated with male infertility^[221]^.

Relative to normozoospermic fertile individuals, patients suffering from oligozoospermia had significantly reduced expression of miR-34b in both semen and testicular biopsies^[222-224]^. Further, microarray data from human semen EVs indicate that miR-21 and miR-148a are underexpressed in men with oligoasthenozoospermia relative to control patient samples^[225]^. These data are in line with small RNA sequencing data from both human and boar semen EVs in which these miRNAs are overexpressed in control samples^[71,72]^.

Proteomic analysis of seminal EVs identified significant differences in expression patterns between asthenozoospermia and normospermia semen samples, and notably decreased expression of ADAM7 and TRPV6, which modulate sperm motility^[226]^. 

Azoospermia is typically categorized as obstructive or nonobstructive; nonobstructive implies that the testes suffer from decreased sperm production. Patients with nonobstructive azoospermia can undergo sperm retrieval procedures, but this is successful in just over half of cases and runs the risk of severe complications^[227,228]^. Seminal EV long-noncoding RNAs may be indicative of promising candidates for sperm retrieval by revealing whether viable sperm are present in the testes^[229]^. Similarly, seminal EV miR-31-5p may be predictive of azoospermia, and miR-539-5p and miR-941 may indicate whether residual sperm is present in the testes or not^[230]^. New approaches to treat nonobstructive azoospermia are emerging, utilizing EVs derived from mesenchymal stem cells or amniotic fluid, in hopes of restoring spermatogenesis^[231,232]^.

#### Female infertility

Although much progress has been made in treating infertility, women with polycystic ovarian syndrome (PCOS), intrauterine adhesions (IUA), or premature ovarian insufficiency (POI) are still struggling to conceive^[233,234]^. EVs may participate in the pathogenesis of these disorders and may also be utilized as therapeutic agents, specifically mesenchymal stem cell derived EVs (MSC-EVs) as they exhibit higher biological stability and lower immunogenicity than traditional MSC cell-based therapy^[235]^.

PCOS is the most common cause of female infertility and is characterized by high levels of androgens, which inhibit normal oocyte development and release. Expression of several miRNAs in the FF of PCOS patients, including miR-132 and miR-320, were significantly lower than in controls; these miRNAs target genes associated with steroidogenesis which is typically impacted in PCOS^[84]^. Proteomic profiling of FF-EVs from PCOS patients revealed enrichment of S100 calcium-binding protein A9, which disrupts steroidogenesis and activates the NF-kB signaling pathway, therefore inducing inflammation^[236]^.

Endometriosis is another common cause of sub-fertility characterized by endometrial tissue developing outside of the uterus. Recently, uterosomes have begun to be investigated for use as biomarkers for endometriosis and patients diagnosed with endometriosis have significantly more circulating EVs relative to control patients^[237,238]^. Vaginal EVs may also serve as non-invasive, early biomarkers of endometriosis as evidence from nonhuman primates indicates that there are fewer EVs in cervicovaginal fluids from a rhesus macaque diagnosed with endometriosis relative to healthy controls^[239]^. Unique to patients diagnosed with endometriosis, plasma EVs contain miR-30d-5p, miR-16-5p, and miR-27a-3p, all of which have previously been associated with endometriosis^[240]^. Further, endometriosis EVs have also been shown to express unique long noncoding RNA (lncRNA) and proteomic profiles that may transfer inflammatory and angiogenic factors to endothelial epithelial cells^[240,241]^.

EVs isolated from cultured endometrial stromal cells biopsied from patients with endometriosis had reduced expression of miR-214, which targets the fibrotic markers connective tissue growth factor (CTGF) and collagen aI^[242]^. Unregulated expression of CTGF and collagen may be involved in the development of endometrial tissue outside of the uterus. Additionally, another group found that EVs isolated from endometriosis patients affected immune and angiogenic factors within the uterine microenvironment^[240]^. EVs derived from primary endometrial cells cultured from a mouse model of endometriosis were shown to be taken up by macrophages both *in vitro *and *in vivo*, attenuate their phagocytic capacity, and induce them to polarize into the M2 phenotype^[243]^. These data suggest that endometriosis-EVs alter the immune microenvironment of the uterus and may contribute to the pathophysiology of the disease. 

Recent studies have shown that MSC-EVs induce angiogenesis in the ovaries of mouse models of chemically induced POI^[244,245]^. Increased ovarian angiogenesis may rescue ovarian function and be an avenue of exploration for treating POI^[244]^. Indeed, rescue of fertility by MSC-EVs has recently been demonstrated, with little to no adverse effects on offspring born to previously infertile mice^[245]^. Aside from angiogenesis, MSC-EVs may also activate the Hippo pathway, which is a key mechanism that mediates folliculogenesis and ovarian function^[246]^.

## CONCLUSION

The role of EVs in reproductive health and pregnancy is an important area of study that is under intense investigation. EVs are key mediators in sperm production and maturation and have been found in every major compartment of the male reproductive system (i.e. the testes, epididymis, seminal vesicles, prostate, and ejaculate). Similarly, in the female reproductive tract, EVs in the ovaries, fallopian tubes, uterus, and vagina have been shown to play significant roles in oocyte development, maturation, and release during the menstrual cycle. Further, EVs from both the male and female reproductive tracts are critical for fertilization and initial development of the embryo, and shortly after fertilization, the embryo itself begins to release EVs that participate in important maternal-fetal crosstalk to ensure proper uterine implantation. As development proceeds, the placenta also begins to release EVs; these EVs are highly abundant in the maternal circulation and likely assist in the development of maternal immune tolerance to the fetus.

In pregnancy related disorders such as preeclampsia, GDM, preterm birth, and fetal growth restriction, EVs have been implicated as direct participants in disease pathophysiology and are being investigated for their use as non-invasive early diagnostic biomarkers. Early identification of pregnant individuals at risk for the development of these complications could facilitate the implementation of life-saving measures to decrease both maternal and fetal mortality. Recent evidence also suggests that the ability to even get pregnant in the first place may rely heavily upon proper EV-mediated signaling pathways. As EVs have been implicated in sex cell development and maturation, it is not surprising that infertility related conditions are characterized by aberrant expression of various proteins and RNAs associated with reproductive tract EVs. 

Understanding the pathophysiology of various reproductive and pregnancy related diseases and conditions will undoubtedly include the investigation of the role EVs play in these conditions. EVs are key functional players in ensuring optimal reproductive health, as well as the initiation and maintenance of successful pregnancies.

## DECLARATIONS

### Copyright

© The Author(s) 2022.
